# Editorial: Mast cells in allergic diseases

**DOI:** 10.3389/falgy.2023.1248954

**Published:** 2023-07-18

**Authors:** Stephanie Kubala, Tamara T. Haque

**Affiliations:** Laboratory of Allergic Disease, National Institute of Allergy and Infectious Diseases (NIH), Bethesda, MD, United States

**Keywords:** allergy, mast cell (MC), IgE (Immunoglobulin E), atopic allergic conditions, FcER1

**Editorial on the Research Topic** Mast cells in allergic diseases

Allergic disease poses a major public health concern that affects 10%–30% of the global population. The etiology of allergic diseases is complex and multifaceted. Although enormous strides have been made in the elucidation and treatment of allergic conditions, many questions remain. Mast cells are sentinel innate immune cells that play a key effector function in the allergic diathesis. Mast cells express the high affinity IgE receptor Fc*ε*RI, which upon IgE and antigen cross-linkage, triggers the release of mediators that are directly responsible for allergic symptomatology. As part of the ongoing effort to demystify allergic inflammation, many studies have focused on the mast cell since its discovery in 1879 by Paul Ehrlich, followed by renewed interest after the discovery of IgE in 1967 by Kimishige Ishikaka. This editorial will summarize recent efforts which have been published in the Research Topic *Mast Cells in Allergic Diseases* in the journal *Frontiers in Allergy*.

**Figure 1:**
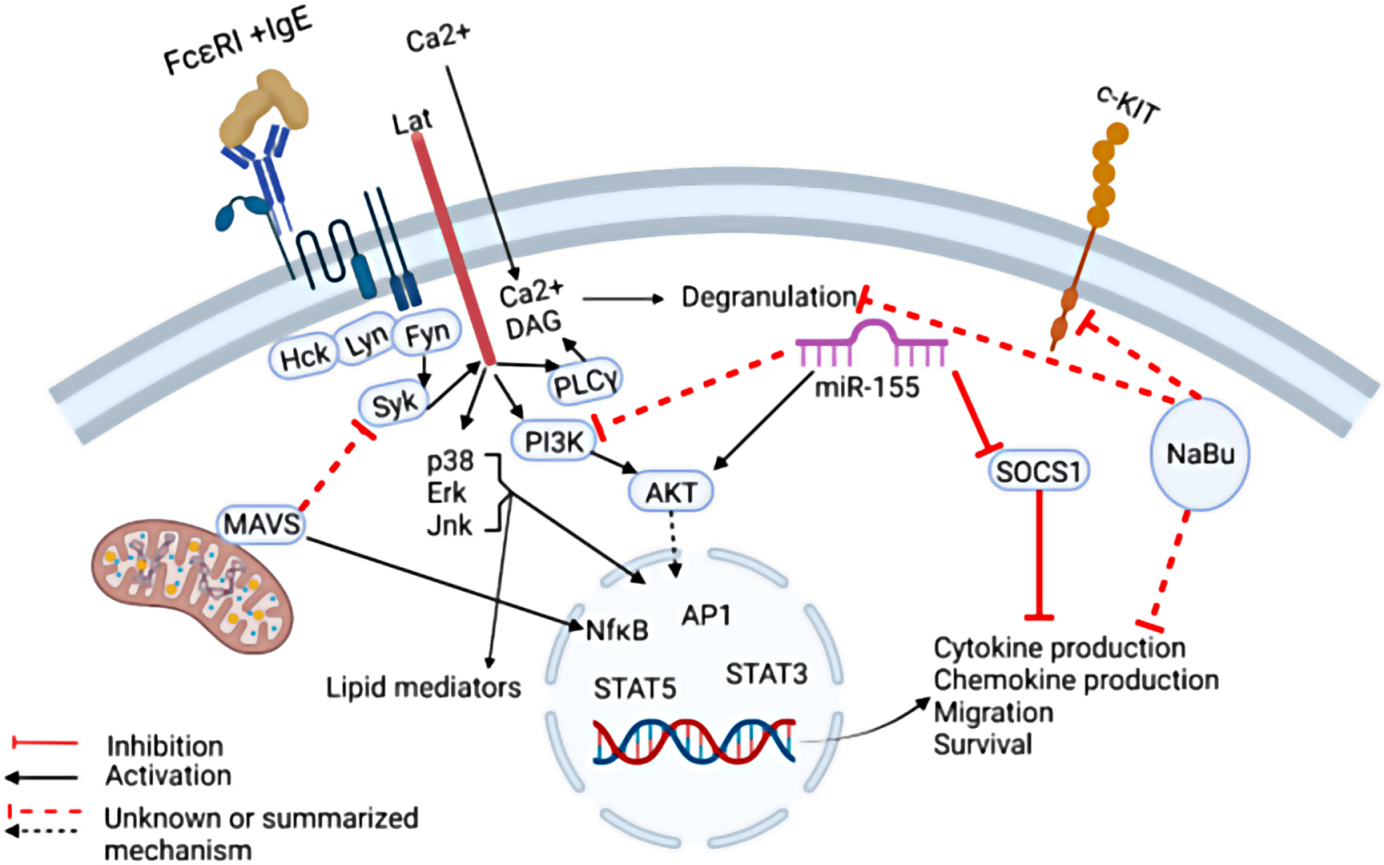
The regulation of IgE-mediated mast cell functions. IgE-mediated cytokine production can be suppressed by Mitochondrial antiviral signaling protein (MAVS) by targeting the signaling molecule SYK. In addition, Sodium Butyrate (NaBu) suppresses mast cell cKIT expression and cytokine production via mechanisms not completely understood. MiR-155 was shown to suppress SOCS1 expression which is a negative regulator of IgE-mediated cytokine production, thereby promoting IgE-mediated cytokine production. In addition, MiR-155 deficiency in mast cells led to increased IgE-mediated cytokine production via enhanced PI3Kγ expression in one study, however in another study, miR-155 deficiency in mast cells led to decreased cytokine production. Taken together, these findings highlight the need for further studies to fully elucidate the complex signals that govern mast cell IgE-mediated functions.

MacDonald et al. demonstrated that sodium butyrate suppresses the cell cycle progression of three malignant human mast cell lines. Butyrate is a short chain fatty acid microbial metabolite that functions as a class I histone deacetylase inhibitor (HDACi). The role of butyrate in promoting gastrointestinal health is well accepted, however its role in allergy remains controversial and unclear. Although multiple murine studies demonstrated that butyrate and butyrate-producing microbes attenuate allergic diseases, human association studies remain conflicting and uncertain, most likely due to the complex function of butyrate in multiple immune cell types (1, 2). Early studies have shown that sodium butyrate enhances mast cell maturation and increases granularity in cultured human and mouse cells, leading to enhanced mast cell degranulation (3–5). Conversely, a recent study demonstrated that butyrate suppressed primary human and murine mast cell IgE-mediated degranulation and IL-6 production (6). MacDonald et al. show that butyrate has an enhancing effect; however, these were “minimal” in the mast cell lines. Furthermore, they demonstrate that butyrate suppresses the expression of Kit (Figure 1) (6). This is potentially important as Kit is the receptor for the mast cell growth factor, SCF, and gain of function mutations in the gene encoding Kit are responsible for most mast cell malignancies. Although the role of sodium butyrate in mast cell mediated diseases remains unclear, these studies provide evidence that it may be an important regulator of mast cells.

Toshiaki Kawakami et al. summarized the role of IgE in the rhinovirus exacerbation of asthma in a prospective review. It is well known that rhinoviruses are associated with asthma exacerbations, and this was recently reviewed by David Jackson and James Gern (7). Clinically, it is clear that IgE plays a role in this association since anti-IgE therapy was shown to reduce infection-associated asthma exacerbations in a randomized clinical trial (8). Mechanistically, multiple hypotheses are plausible as summarized in these two reviews, however uncertainties remain and a complete understanding of the relationship between viral and allergic inflammation requires further investigation. One step towards understanding the role of mast cells in viral immunity was provided by a brief research report by Yuko Kawakami et al. who demonstrated that the mitochondrial antiviral signaling protein (MAVS) plays a negative role in IgE-mediated cytokine production, possibly by altering SYK activation (Figure 1). The implication of this finding requires further *in vivo* studies utilizing viral infection and allergy models.

MicroRNAs (miR) are single stranded noncoding RNA molecules that have been shown to profoundly regulate various physiological processes through post-transcriptional gene regulation, including the immune system. In particular, the association between miR-155 and allergic diseases have been examined in multiple studies. Previously, miR-155 was shown to play both a negative and positive role in IgE-mediated mast cell functions by two independent groups. One group demonstrated that *in vitro* cultured mast cells deficient in miR-155 exhibited stronger IgE-mediated activation via enhanced PI3K*γ* activity (9). Another group demonstrated that IL-10 induced miR-155 enhanced IgE-mediated mast cell functions by suppressing a negative regulator, suppressor of cytokine signaling 1 (SOCS1) (10).

Adding to these discoveries, Mohammed et al. demonstrated that IgE and antigen cross-linking induced miR-155 in human and murine mast cells *in vitro*. Furthermore, using murine bone marrow derived mast cells, the authors showed that miR-155 deficiency resulted in diminished Cyclooxygenase-2 expression as well as cytokine production (Figure 1). The conflicting results may be due to differences in culture conditions, animal sex, or animal facility microbiome. Further studies are needed to dissect the nuance of miR-155 regulation of mast cells and its relationship to allergic diseases.

Taken together, this Research Topic on mast cells in allergic diseases has led to a positive step towards understanding the complex regulation of mast cells via butyrates, anti-viral receptors, and miRs, as summarized in Figure 1.
